# Aberrant interhemispheric functional reciprocities of the default mode network and motor network in subcortical ischemic stroke patients with motor impairment: A longitudinal study

**DOI:** 10.3389/fneur.2022.996621

**Published:** 2022-10-04

**Authors:** Yongxin Li, Zeyun Yu, Xuan Zhou, Ping Wu, Jiaxu Chen

**Affiliations:** ^1^School of Traditional Chinese Medicine, Formula-Pattern Research Center, Jinan University, Guangzhou, China; ^2^Acupuncture and Tuina School/Tird Teaching Hospital, Chengdu University of Traditional Chinese Medicine, Chengdu, China

**Keywords:** subcortical stroke, resting-state functional magnetic resonance imaging, functional homotopy, superior precuneus, machine learning

## Abstract

**Purpose:**

The purpose of the present study was to explore the longitudinal changes in functional homotopy in the default mode network (DMN) and motor network and its relationships with clinical characteristics in patients with stroke.

**Methods:**

Resting-state functional magnetic resonance imaging was performed in stroke patients with subcortical ischemic lesions and healthy controls. The voxel-mirrored homotopic connectivity (VMHC) method was used to examine the differences in functional homotopy in patients with stroke between the two time points. Support vector machine (SVM) and correlation analyses were also applied to investigate whether the detected significant changes in VMHC were the specific feature in patients with stroke.

**Results:**

The patients with stroke had significantly lower VMHC in the DMN and motor-related regions than the controls, including in the precuneus, parahippocampus, precentral gyrus, supplementary motor area, and middle frontal gyrus. Longitudinal analysis revealed that the impaired VMHC of the superior precuneus showed a significant increase at the second time point, which was no longer significantly different from the controls. Between the two time points, the changes in VMHC in the superior precuneus were significantly correlated with the changes in clinical scores. SVM analysis revealed that the VMHC of the superior precuneus could be used to correctly identify the patients with stroke from the controls with a statistically significant accuracy of 81.25% (*P* ≤ 0.003).

**Conclusions:**

Our findings indicated that the increased VMHC in the superior precuneus could be regarded as the neuroimaging manifestation of functional recovery. The significant correlation and the discriminative power in classification results might provide novel evidence to understand the neural mechanisms responsible for brain reorganization after stroke.

## Introduction

Subcortical ischemic stroke is a common acute cerebrovascular disease, with approximately half of the patients exhibiting residual disabilities (1). Because of cerebral thrombosis or cerebral embolism in the brain, brain dysfunction always occurs within the default mode network (DMN) and motor-related areas, such as the precuneus, parahippocampus, supplementary motor area (SMA), primary motor cortex (M1), and premotor cortex (2–6). The DMN is one of the most widely studied functional brain networks that support aspects of cognition. Patients' cognitive recovery after stroke has been reported to be associated with functional connectivity (FC) impairments within the DMN (4, 7). The precuneus is a key region of the DMN. Recently, several studies have reported that patients with ischemic stroke exhibited decreased connectivity in the precuneus (8–10). Because of the important role of the precuneus in a wide spectrum of higher-order brain functions, such as motor function, decreased connectivity of the precuneus could inhibit the activity in the motor cortex and impact patients' motor function. It was reported that ~50% of patients with strokes showed impaired hand motor function in the chronic phase, which always caused negative effects on the quality of life (11). Additionally, brain function recovery after stroke is always difficult to evaluate (12). Mounting evidence has suggested that the recovery of impaired behavioral function is accompanied by brain reorganization after effective treatment (13–17). Identifying stroke recovery processes by neuroimaging will benefit the selection of suitable treatments for stroke.

Neuroimaging has become the most useful tool available to detect the functional and structural organization of the human brain. Previous neuroimaging studies have extensively explored the changes in the DMN and motor-related networks after stroke (4, 18–21). These studies have shown system-wide network disturbances and brain functional reorganization in patients with stroke. Additionally, analyses of functional and structural connectivity were also used to investigate the effects of interventions on the brain network following stroke (13, 15, 22). For example, a previous study found that patients with stroke showed a significant decrease in FC between the bilateral M1 after stroke, and the disrupted FC was restored with antiplatelet therapy (22). Patients with chronic stroke completed a rehabilitation protocol and showed significantly enhanced FC between the posterior cingulate cortex (PCC)/precuneus and M1 in contrast to the prerehabilitation condition (16). The long-term training effect following robot-hand training was also detected in patients after stroke (23). A recent study on patients with stroke found that brain-computer interface technology could significantly improve interhemispheric FC in the motor network and motor outcomes poststroke (24). These previous studies implied that brain function recovery in patients with stroke is attributed to brain reorganization (25).

A model called functional homotopy was developed based on resting-state functional magnetic resonance imaging (MRI) to directly quantify interhemispheric functional reciprocities (19, 26). In this model, the brain shows a high degree of synchrony in patterns of spontaneous activity between homotopic interhemispheric counterparts (geometrically corresponding). Voxel-mirrored homotopic connectivity (VMHC) is a validated method that can be used to quantify functional homotopy between two hemispheres (27). VMHC is one of the most noticeable characteristics of the brain's essential functional architecture, which has an important influence on cognition and behavior by interhemispheric communication. Recently, as a conspicuous indicator of disturbed functional specialization of the brain, VMHC has been chosen in some diseases to explore the alterations of functional homotopy due to intervention or recovery (28–31). This method has also been used in stroke studies to investigate the changes in functional connections between cerebral hemispheres (10, 17, 28, 31). After receiving scalp acupuncture, patients with acute ischemic stroke showed a significant increase in VMHC values in the bilateral BA6 and BA8 (17). Scalp acupuncture can specifically strengthen the functional activities of the brain regions related to motor coordination in stroke. A recent study revealed that synchrony between the DMN and the sensorimotor network can facilitate motor recovery after stroke rehabilitation (16). Although the connectivity between the DMN and motor-related regions has been investigated in the above previous stroke studies, the longitudinal changes of the interhemispheric connection have neither been considered in stroke studies nor have the relevance between the changes in interhemispheric connection in these networks and the changes in clinical scores.

Thus, the purpose of the present study was to elucidate the neurological mechanism of the DMN and the motor network organization process in stroke, which has attracted intense attention from clinical and basic researchers. The VMHC method can be used to quantify interhemispheric connectivity. The longitudinal changes in functional homotopy in these networks provide some insights into the mechanism underlying recovery processing in patients with stroke. In order to achieve these purposes, we detected the VMHC changes in patients with subcortical ischemic stroke with motor impairment, hypothesizing that patients with ischemic stroke would show reduced interhemispheric functional reciprocities. Given the evidence for interhemispheric functional and structural pathway dysfunction in patients with stroke (17, 32–35), we expected the DMN and motor-related areas to be particularly affected. In patients with stroke, the abnormal VMHC partially recovered after treatment, and the affected VMHC between the bilateral hemispheres may be related to behavioral performance. Furthermore, we used a machine learning approach to assess whether these group differences could be used to accurately distinguish patients with stroke from healthy controls.

## Methods and materials

### Participants

Nineteen first-ever patients with stroke (eight women; mean age ± SD: 64.7 ± 12.4 years; range from 37 to 81 years old) with unimanual motion defects due to subcortical ischemic lesions were recruited from the Department of Neurology of the First Affiliated Hospital of the Chengdu University of Traditional Chinese Medicine, China. Most of the lesions were located in the basal ganglia and nearby regions. The demographic data and clinical characteristics of patients with stroke are provided in Table 1. The inclusion criteria for patients were as follows: (1) pure unilateral motor hemiparesis at least 20 days after the first onset ischemic stroke incident (confirmation and location of stroke by MRI); (2) no other white matter pathology as proven by structural MRI; (3) absence of additional psychiatric or neurologic disorders; (4) no neglect, aphasia or dementia; and (5) no other previous experimental therapy before participating in this research. Thirteen age-matched healthy subjects (eight females; mean age ± SD: 62.1 ± 10.8 years) served as controls. Control subjects did not exhibit any history of medical disorders and were not taking regular medication. All participants gave written informed consent before the study. This study was approved by the Ethics Committee of Chengdu University of Traditional Chinese Medicine (no. 2011KL-002), and this method was carried out in consideration of the approved guidelines.

**Table 1:** Demographical and clinical data.

**Patient number**	**Sex**	**Age (years)**	**Affected hand**	**Pathogeny**	**Lesion duration (days)**	**FMA pre**	**FMA post**	**NDS pre**	**NDS post**
1	F	69	Right	Left pons-centrum semiovale	135	90	96	13	5
2	M	73	Right	Left basal ganglia and centrum semiovale	132	80	84	26	23
3	F	43	Right	Left caudate nucleus and periventricular	25	92	96	15	6
4	F	71	Left	Right basal ganglia	32	88	95	21	10
5	F	75	Right	Left occipital gyrus and thalamus	22	74	90	32	17
6	M	73	Right	Left basal ganglia-centrum semiovale	26	84	91	24	18
7	M	81	Right	Left basal ganglia-internal capsule	22	83	92	23	12
8	M	49	Right	Left basal ganglia and centrum semiovale	56	85	93	24	16
9	M	67	Right	Left basal ganglia and periventricular	33	80	93	26	12
10	M	78	Right	Left basal ganglia-posterior horn of lateral ventricle	21	84	95	19	9
11	F	70	Right	Left centrum semiovale-periventricular	23	85	90	24	2
12	F	74	Right	Left periventricular	148	90	96	20	12
13	M	37	Right	Left thalamus-lenticular nucleus	148	88	94	24	14
14	M	79	Left	Right basal ganglia	50	85	92	26	15
15	F	60	Right	Left basal ganglia and radial area	23	80	85	28	20
16	M	73	Right	Left capsula externa-periventricular	56	77	90	31	20
17	F	54	Right	Left basal ganglia	45	80	85	26	23
18	F	54	Right	Left basal ganglia	23	86	92	22	14
19	M	63	Right	Left thalamus	21	82	85	25	18

F, female; FMA, Fugl-Meyer Motor Assessment; L, left; M, male; MBI, Modified Barthel Index; R, right.

### Treatment and behavioral evaluation of recovery

Antiplatelet therapy was administered to all patients with stroke (10 mg Erigeron breviscapus injection, 75 mg clopidogrel once each day taken orally). Then, citicoline (0.5 g, daily) was injected intravenously to improve the clinical effects. Drug therapy was conducted for 1 month (30 days) for each patient. The functional MRI data of each patient were collected before and after the treatment at ~1-month intervals. The clinical evaluations of recovery, such as the Fugl-Meyer Assessment (FMA) and the Neurological Deficit Scores (NDS), were carried out on the same days when we collected the functional MRI scans of the patients. The FMA is one of the most established and commonly used motor outcome measures in stroke rehabilitative trials (36). This scale includes a total of 50 items, such as assessments of the tendon reflex; joint movement of the flexor and extensor of the shoulder; elbow, wrist, knee, and hip joints; the movement of small joints with the stability of the wrist and the ankle; and coordination ability and speed. The highest total score is 100 points, and the higher the scores are, the milder the impairments in motor function. As a trial outcome measure in patients with stroke, the NDS was performed to the severity of neurological functional deficits and to assess the severity of strokes.

### Data acquisition

All fMRI data were acquired with a 3-T Siemens scanner (MAGNETOM Trio Tim, Siemens, Erlangen, Germany) at the West China Hospital MRI Center. We used a gradient echo planar imaging sequence to acquire fMRI data: 30 interleaved axial slices, slice thickness = 5 mm, repetition time (TR) = 2,000 ms, echo time (TE) = 30 ms, flip angle = 90°, field of view = 240 mm × 240 mm, matrix = 64 × 64, and 180 volumes. The three-dimensional T1-weighted structural MRI was collected using a spin-echo planar image sequence with the following parameters: slices = 176; TR/TE = 1,900 /2.26 ms, flip angle = 9°; field of view = 256 × 256 mm; voxel size = 1 × 1 × 1 mm^3^. During the scanning, the head of each participant was fixed using foam cushions to limit head movement. All participants were instructed to remain awake, relax with their eyes closed, remain motionless, and try not to think about anything.

**Figure 1:**
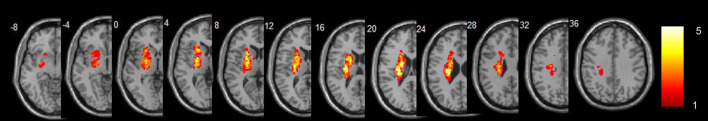
Lesion overlay map displaying regions of lesion overlap between participants. The *n*-value denotes the number of patients with a lesion in each voxel. L, left.

The lesions were manually segmented using MRIcron software on the T1-weighted MRI images. We generated a lesion mask for each patient and normalized the results to the Montreal Neurologic Institute (MNI) space. The lesion overlay map is displayed in Figure 1. The lesions of the patients were mainly located in the left basal ganglia.

### Imaging processing and statistical analysis

#### Behavioral data analysis

A paired *t*-test was conducted to examine whether the patients with stroke had actually improved based on the clinical scores from pretreatment to posttreatment.

#### Resting state functional connectivity

Data processing was conducted using the statistical parametric mapping (SPM12, London, UK, http://www.fil.ion.ucl.ac.uk/spm) package. The first 10 volumes of each subject were discarded in consideration of magnetization equilibrium effects and the adaptation of the subjects to the circumstances. Preprocessing comprised the following steps: (1) slice time correction; (2) head motion correction; (3) coregistration of the individual T1-weighted images to functional images; (4) segmentation of the T1-weighted images (gray matter, white matter, and cerebrospinal fluid) and spatial normalization to the MNI space by using a 12-parameter nonlinear transformation; (5) application of the transformation parameters to the functional images; (6) resampling to a voxel size of 3 × 3 × 3 mm^3^; (7) smoothing (full width at half maximum =6 mm); (8) temporal bandpass filtering (0.01–0.08 Hz); (9) linear and quadratic detrending; (10) regression of several nuisance covariates (white matter, cerebrospinal fluid, global signal, and the Friston 24 head motion parameters).

In the present study, we mainly focused on the DMN and motor network, which is remote from the primary lesion. We selected a user-defined mask including the DMN and motor network for the following analysis. This mask was produced from the cortical parcellation maps of the Yeo2011 resting state network (https://surfer.nmr.mgh.harvard.edu/fswiki/CorticalParcellation_Yeo2011). The components of the DMN and motor network in this template were extracted and combined to produce the user-defined mask. Then, this mask was averaged with its left-right mirrored version to generate a symmetrical mask for the following VMHC analysis (refer to Supplementary Figure 1 for the symmetrical mask).

### Voxel-mirrored homotopic connectivity

The VMHC was calculated using the DPARSF (http://resting-fmri.sourceforge.net) toolbox (37). For each subject, the Pearson correlation between each pair of mirrored voxel time series was computed to regard homotopic connectivity (27). Fisher z-transformation was then performed on the correlation values to improve normality and generate VMHC maps. Two-sample *t*- test analysis was performed to compare the VMHC map differences between the patients and the controls in the mask. Paired *t*-test analysis was performed to assess the longitudinal changes in patients between the two time points. The threshold of the resulting statistical map was a combination of *p* < 0.005 for a single voxel and a minimum cluster size of 297 mm^3^, which corresponds to a corrected threshold of *p* < 0.05 (AlphaSim correction). During the statistical analyses, age and sex were modeled as covariates. Finally, the regions that showed significant differences between groups were selected as our regions of interest, and the mean VMHC values were extracted from these regions for the following analyses. Treatment effects on the VMHC of these interesting regions were calculated among these groups.

### Correlation between the neuroimaging index and clinical scores

To assess the clinical relevance of altered homotopic FC, partial correlations were calculated between the change in VMHC and the change in each clinical score between the two time points in patients with stroke. The FMA and NDS scores were included as analyzed clinical variables. The threshold was set at *p* < 0.05. The correlation results were also corrected by the Bonferroni method based on the number of regions selected as regions of interest. During the partial correlations, analyses were controlled for age, sex, disease duration, and lesion sizes.

### Support vector machine analysis

To detect the above group differences specific to the patient group, a support vector machine analysis (SVM) was performed on neuroimaging data to extract patterns and clarify the patterns in patients with stroke and healthy controls (38, 39). The SVM was implemented using the Pattern Recognition for Neuroimaging Toolbox (PRoNTo) software (http://www.mlnl.cs.ucl.ac.uk/pronto). Individual resting-state fMRI data were treated as points located in a high dimensional space defined by the VMHC values in the preprocessed images. The regions showing significant correlation were selected as masks. The mask was applied to each image to identify the VMHC values as a feature in the model. A binary SVM machine was used as the classifier in the present study (i.e., patients with stroke (pretreatment) vs. healthy controls). Due to our limited number of samples, a “leave-one-out” method was used during the cross-validation step (38). Much of machine learning theory rests on the assumption that the data are independently and identically distributed. For functional neuroimaging, the data are within-run correlations and hemodynamic effects, which often do not meet the above assumption. To apply machine learning theory to functional neuroimaging data, permutation testing is needed, which enables us to obtain *p*-values for the performance metrics. When the SVM algorithm was established, permutation tests (repeated 1,000 times) were used to evaluate the performance of the SVM model. Finally, we obtained the corresponding accuracy, sensitivity, specificity, and area under the receiver operating characteristic curve.

## Results

### Behavioral data

No significant differences were observed in sex (χ^2^ = 1.17, *p* = 0.28) or age (*t* = 0.24, *p* = 0.81) between the patients and the healthy controls. With a 1-month interval, the FMA score was significantly increased (pair T: *t* = −10.077, *p* < 0.001) from 84.3 ± 4.1 (first time point) to 91.5 ± 4.1 (second time point). The NDS scores also showed a significant decrease (pair T: *t* = 13.0, *p* < 0.001) from 23.3 ± 4.3 (first time point) to 14.2 ± 5.2 (second time point).

### VMHC: Group differences

**Figure 2:**
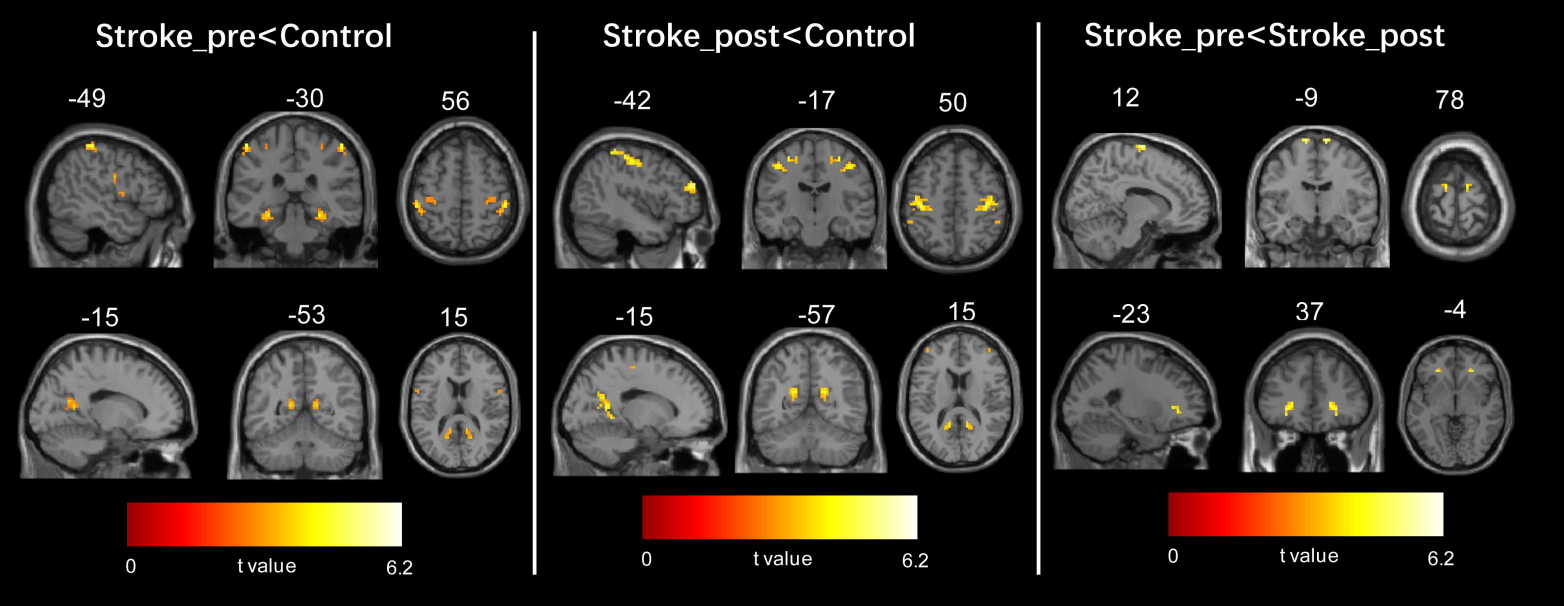
Regions showing significant changes in voxel-mirrored homotopic connectivity (VMHC) between each pair of the three groups. The threshold of the resulting statistical map was a combination of *p* < 0.005 for a single voxel and a minimum cluster size of 297 mm^3^.

The patients with stroke at the first timepoint showed a significantly lower VMHC in some regions than the healthy controls, such as in the parahippocampus, precuneus, precentral gyrus, and paracentral lobule/SMA (refer to Figure 2 left panel and Table 2). No areas showed a significant increase in VMHC in patients with stroke at the first time point.

**Table 2:** Significant group differences in VMHC.

**Cluster location**	**Statistical values**		**Peak (MNI)**		
	**Cluster size**	***t*-value**	**x**	**y**	**z**
**Control** **>** **Patient_pre**					
Hippocampus/Fusiform	25	4.52	±45	−27	−18
Parahippocampus	21	4.44	±27	−30	−15
Middle temporal gyrus	43	3.75	±57	−51	−6
Middle temporal gyrus	58	4.47	±51	−66	12
Precentral gyrus	124	4.36	±60	3	24
Postcentral gyrus		4.07	±51	−9	27
Precuneus	30	3.92	±12	−51	12
Postcentral gyrus	27	3.40	±42	−21	48
Superior parietal lobe	36	6.58	±51	−33	60
Paracentral lobule/SMA	11	3.86	±12	−12	78
Superior precuneus	18	5.30	±12	−66	42
**Control** **>** **Patient_post**					
Parahippocampus/Fusiform	20	4.01	±33	−54	−18
Precuneus	64	4.17	±12	−63	24
Inferior frontal gyrus	55	4.46	±57	21	12
Middle frontal gyrus	37	4.65	±42	48	24
Inferior parietal lobe	32	3.84	±66	−27	30
Superior parietal lobe	208	5.37	±51	−33	60
Postcentral gyrus		4.46	±39	−24	51
Precentral gyrus		4.46	±30	−15	60
Precentral gyrus	13	3.55	±48	−6	42
**Patient_pre** **<** **Patient_post**					
SMA	14	5.51	±12	−9	78
Middle frontal gyrus	15	5.48	±23	37	−4

The MNI coordinates and t-values for the local maxima of the centers of the voxel clusters. The threshold for significant clusters reported here was set at p < 0.005 and a cluster size of 297 mm^3^.

VMHC, voxel-mirrored homotopic connectivity; MNI, Montreal Neurological Institute, SMA, supplementary motor area.

The patients with stroke at the second time point still showed a significantly lower VMHC in some regions than the healthy controls, such as the precuneus, precentral gyrus, middle frontal gyrus (MFG), and parahippocampus (refer to Figure 2 middle panel and Table 2). No areas exhibited a significant increase in VMHC in patients with stroke at the second time point.

In patients with stroke, a paired *t*-test to assess the difference between the two time points revealed that the VMHC values in the SMA and MFG significantly increased from the first to the second time point (refer to Figure 2 right panel and Table 2). No areas showed a significant decrease in VMHC in patients with stroke from the first to the second time point.

**Figure 3:**
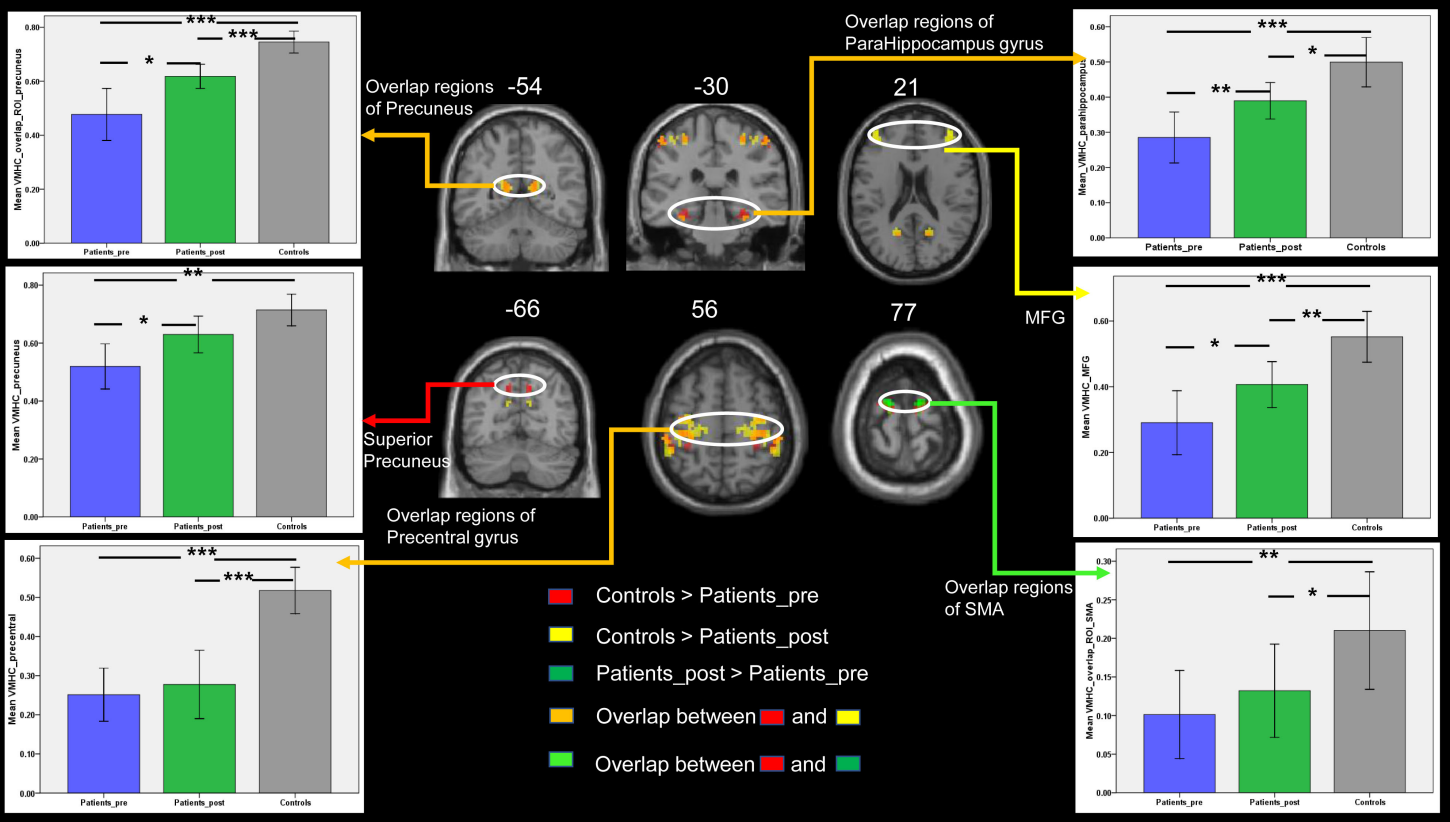
The comparisons of VMHC between the three groups in the DMN and motor-related regions of interest. Bars represent the mean and error bars represent the standard deviations. SMA, supplementary motor area; MFG, middle frontal gyrus. Patients_pre = the patient group in the pretreatment stage. Patients_post = the patient group in the posttreatment stage. *, *p* < 0.05; **, *p* < 0.01; ***, *p* < 0.001.

In the present study, we focused on the regions belonging to the DMN and motor network. Thus, the significant results from the above *t*-test, including the results regarding the bilateral precuneus, parahippocampus, MFG, SMA, and precentral gyrus, were selected as regions of interest in the following analyses. If the above regions of interest were identified in at least two comparison results, we selected the overlap regions as our areas of interest. The mean VMHC values were extracted from each region of interest. Although the disrupted interhemispheric communication measured by VMHC in patients was increased in these interesting regions from the first to the second timepoint, the VMHC in these regions except for the superior precuneus of the patients after treatment, was still significantly lower than that of the controls (Figure 3). Regarding the interesting regions of the superior precuneus, a significant increase in VMHC values was observed in patients with stroke from the first to the second time point. The mean VMHC values in the superior precuneus of the patients at the second time point showed no significant difference from the values in the controls. This result is consistent with the VMHC changes in voxels, as listed in Table 2. The superior precuneus no longer showed a significant difference between the controls and the patients at the second time point. By combining the data regarding the consistent changes in the superior precuneus shown in Table 2 and Figure 3, we selected this region as the key region in the following correlation analysis and SVM analysis.

### Brain–behavior relationships: Correlations between VMHC and FMA or MBI

**Figure 4:**
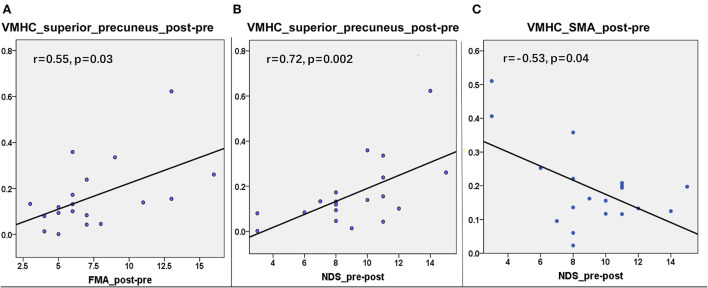
The correlations between the changes in VMHC and the changes in clinical variables from the first timepoint and the second timepoint in the superior precuneus of stroke patients. **(A)** The correlation (*r* = 0.55, *p* = 0.03) between the changes of VMHC in superior precuneus and the changes of FMA. **(B)** The correlation (*r* = 0.72, *p* = 0.002) between the changes of VMHC in superior precuneus and the changes of NDS. **(C)** The correlation (*r* = −0.53, *p* = 0.04) between the changes of VMHC in SMA and the changes of NDS.

To assess whether the VMHC values in the superior precuneus could reflect the motor ability and neurological deficit of patients with stroke, we further explored the relationship between the change in the behavioral scale and the change in the mean VMHC in this region from the first to the second time point. The same correlation processes were performed on the other interesting regions, including the parahippocampus, MFG, precuneus, precentral gyrus, and SMA. The same correlation process was performed six times (six interesting regions) for each behavioral score. The corrected *p*-value of Bonferroni correction on the correlation result was set at 0.0083 (0.05/6). A partial correlation between the changes in VMHC values in the superior precuneus and the change in FMA scores showed a significant correlation in the patient group (*r* = 0.55, *p* = 0.03, refer to Figure 4A). Moreover, the changes in VMHC values in the superior precuneus showed a significant partial correlation with the change in NDS scores (*r* = 0.72, *p* = 0.002, refer to Figure 4B). The changes in VMHC values in the SMA also showed a significant partial correlation with the change of NDS scores (*r* = −0.53, *p* = 0.04, refer to Figure 4C). Analyses were controlled for age, sex, disease duration, and lesion sizes. No other significant correlations were detected in the present study. Only the correlation between the change in VMHC values in the superior precuneus and the change in NDS scores could withstand the Bonferroni correction (*p* < 0.0083).

### SVM classification

**Figure 5:**
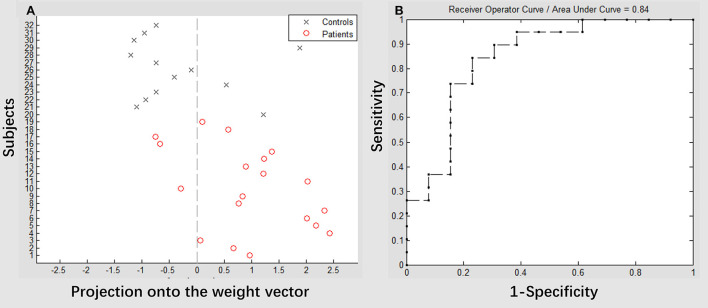
Classification plot **(A)** and receiver operating characteristic (ROC) curve **(B)** for the comparison between groups. Voxel-mirrored homotopic connectivity maps of 19 patients with stroke (pretreatment) and 13 healthy controls were used for classification, which yielded an accuracy of 81.25% (84.21% sensitivity, 76.92% specificity), which was statistically significant at *p* < 0.003.

The result of the SVM classification between 19 patients with stroke and 13 healthy controls based on the feature of VMHC in the superior precuneus derived from resting-state fMRI can be seen in Figure 5. This SVM classifier achieved an accuracy of 81.25% (*p* < 0.003). The model exhibited a sensitivity of 84.21% and specificity of 76.92%. The area under the receiver operating characteristic curve (AUC) value was 0.84. We also used the binary SVM machine based on the VMHC features in the superior precuneus to classify patients with stroke (posttreatment) and healthy controls. The analysis of SVM classification achieved an accuracy of 31.25%, which was statistically significant at *p* < 0.953 (refer to Supplementary Figure 2).

## Discussion

The present study investigated the longitudinal changes in VMHC in the DMN and motor-related regions of patients with subcortical stroke between two time points with a 1-month interval. Patients with stroke showed a significantly lower VMHC in the precuneus, parahippocampus, MFG, SMA, and precentral gyrus than the healthy controls. Although the impaired VMHC in these regions was enhanced after 1 month of treatment, the VMHC values in all but the superior precuneus were still significantly lower than those in the healthy controls. The decreased VMHC values in the superior precuneus of the patients were enhanced significantly at the second time point and showed no significant difference from those in the healthy controls. Furthermore, between the two time points, the changes in VMHC in the superior precuneus region were significantly related to the changes in clinical scores in patients with stroke. The SVM analysis results demonstrated that the VMHC values in the superior precuneus exhibited discriminative power in the classification of patients with stroke from healthy controls. The recovery of functional homotopy and the discriminative power in the classification of the superior precuneus belonging to the DMN may provide novel evidence for understanding the neural mechanisms responsible for brain reorganization in stroke.

### Significant changes in VMHC in patients with stroke

In the present study, patients with stroke showed a significant decrease in VMHC in the precuneus, parahippocampus, and MFG, which are part of the DMN. The DMN is one of the most widely studied functional brain networks at rest (40). This network shows reduced activity during tasks and presents higher levels of activity than other networks during rest periods. The DMN plays an important role in “resting” brain activity, which is involved in emotional control, self-consciousness, and sustaining attention (41). Previous resting-state functional studies in stroke showed that DMN disruption is a common observation (20, 42, 43). Patients with stroke showed lower activity in the DMN and lower functional connectivity between the precuneus and other regions than healthy controls (7, 9, 44). The present study found decreased functional homotopy in the DMN in patients with stroke, which is similar to these previous fMRI studies. Reductions in DMN activity and connectivity can disrupt the cognitive processes mediated by the associated network. A previous study showed that the coordination between two hemispheres plays a significant role in human behavior (45). The decreased VMHC in the DMN that was observed in the present study indicated that disrupted interhemispheric connectivity may have some correlations with the occurrence of cognitive impairment in patients with stroke. The precuneus is the posterior region of the superior parietal lobe. This region plays an important role in the implementation of higher-order brain functions. Disruption of the precuneus can be considered an early sign of cognitive impairment (8). The precuneus has also been proposed as a structural and functional hub in the brain connectome. A previous study also used the VMHC method in patients with stroke and observed that patients showed significantly decreased VMHC values in the bilateral precuneus and precentral gyrus (10). Thus, the decreased functional homotopy between the bilateral precuneus observed in the present study was consistent with the results of a previous study, and this result can be regarded as the neuroimaging manifestation of cognitive disturbance in stroke.

Numerous studies on stroke have also reported disruptions in FC in the cortical motor-related network after stroke. For instance, previous studies have demonstrated that stroke can cause significant disturbances in the effective connectivity of motor areas (5, 14, 18). FC between the bilateral primary sensorimotor cortex was significantly decreased in patients with stroke (17, 46). Using diffusion tensor imaging (DTI), patients with stroke also demonstrated a decrease in the interhemispheric fiber connections between the left and right motor cortex (33, 47, 48). The DTI findings reflect anatomical disconnection, which may underlie the impaired interhemispheric resting-state FC. In the present study, patients with stroke showed significantly lower VMHC in the SMA and precentral gyrus than healthy controls. This result is consistent with previous neuroimaging studies on stroke showing that cortical motor connectivity can reflect poststroke sensorimotor signal processing (5, 49, 50). The SMA has strong anatomical connections with the areas of the central nervous system, including the thalamus, spinal cord, dorsal premotor cortex, and contralateral hemisphere regions (51, 52). In stroke patients with subcortical injuries, the functional ability of the brain enabled by exchanges and cooperation between the two sides of the hemisphere is affected, and motor function is also influenced (49). Thus, it is easy to understand the result that the intrinsic neural interhemispheric coupling of bilateral SMAs was significantly reduced in the patient group. The precentral gyrus and anterior paracentral lobules together form the first somatic movement area, which is the main functional area governing the contralateral somatic movement. A previous DTI study tracing the fibers originating from the precentral gyrus in chronic stroke found that the integrity of all motor tracts showed a descending trend in patients (53). The motor tract damage from the precentral gyrus of patients with stroke can explain the significant decrease of VMHC in the precentral gyrus. Additionally, a consistent result of previous VMHC studies on stroke is that patients with stroke showed a significant decrease in VMHC values between the bilateral precentral gyrus (10, 28, 31). This result observed in the present study is consistent with those of these previous studies. However, the precentral gyrus is the main functional area of contralateral limb movement. The somatomotor center is located in the precentral gyrus, which is the higher center that controls the movement of the body. This region is responsible for regulating and connecting the lower motor center located in the brainstem and spinal cord. Therefore, limb movement disorders after stroke may result from disrupted connectivity of the precentral gyrus.

### Longitudinal changes in VMHC between two time points with a 1-month interval

The most substantial discovery observed in the present research was the longitudinal changes in VMHC in the DMN and motor-related regions of patients with stroke. The functional couplings between the bilateral DMN and motor-related regions were improved. These results are consistent with previous studies showing that the intervention effects of brain networks in patients with stroke were mainly expressed as restorations in the connectivity pattern of interhemispheric interactions and the recovery of cognitive functions (13, 18, 23, 54). Stroke has been reported to be associated with FC impairment within DMN. Significant decreases in FC among the medial frontal cortex, PCC, and precuneus were found in patients with stroke (9, 42). A previous study showed that significantly reduced FC could be detected in the left precuneus, right SMA, and right superior frontal gyrus in patients with basal ganglia stroke (6). In the present study, most patients with stroke had a lesion in the basal ganglia. Thus, aberrant VMHC between the bilateral hubs within the DMN observed in our results can provide information for imaging diagnosis and early intervention. Additionally, the decreased VMHC in the regions of the DMN was enhanced significantly in all patients with stroke after 1 month of treatment. The explanation of this result should combine the functional role of these regions of the DMN in stroke. The precuneus is the intermediate connector between the two hemispheres. Previous studies have revealed that the FC of the PCC/precuneus was increased significantly after a period of rehabilitation treatments or acupuncture treatment (16, 55). Thus, the increased VMHC between the bilateral hemisphere regions within the DMN observed in our study could be interpreted as the effects of the enhancement of brain function recovery.

In addition to the DMN, the VMHC in the SMA and precentral gyrus showed an increase after treatment. All of these regions are involved in motor function. A previous study reported an increase in coupling between the SMA, primary motor cortex, and premotor cortex following rehabilitation (56). These regions may play a critical role in motor recovery. In a resting-state fMRI study of patients with stroke with 3 weeks of upper limb rehabilitation therapy, Jame (57) found a stronger influence of the ipsilesional dorsal premotor cortex on its contralesional homolog. A previous study found that patients with stroke showed a significant increase in VMHC values between the bilateral premotor area and SMA after scalp acupuncture treatment (17). Enhanced interhemispheric communication was associated with improvements in motor performance. One of our previous multimodal neuroimaging studies also revealed that the impaired interhemisphere FC was restored after treatment (5). Patients with stroke also exhibited recovery of the impaired structural connectivity of the corpus callosum following the treatment intervention. In the present study, all patients were treated with 1-month antiplatelet therapy. Patients' clinical scores of FMA and NDS were changed significantly after treatment. The present neuroimaging findings were inline with the behavioral results. Enhanced interhemispheric communication of the SMA and precentral gyrus after treatment in patients with stroke may be the neuroimaging expression of their motor function recovery. Such increased interhemispheric coupling in patients with stroke might be induced by growth-related neurobiological processes enabling the formation of new synapses (58). Based on these results, we can see that the recovery of motor function may depend on reorganization processes within both hemispheres leading to enhanced interhemispheric connectivity (25).

Although the levels of VMHC in the DMN and motor-related regions were increased after antiplatelet therapy in patients with stroke, they were still significantly lower than those in healthy controls. A previous study with a longitudinal design revealed that the neural activity and FC of motor-related areas in patients with stroke returned to normal levels over 1 year after stroke onset (3). The recovery process of the inter-region coupling in patients with subcortical stroke demonstrated a dynamic change following long-term observation (46). The restored VMHC results observed in the present study implied that antiplatelet therapy is efficient in the rehabilitation process of patients with stroke. However, the treatment cycle of 1 month is too short to achieve full recovery of functional connections between the bilateral hemispheres in patients with stroke. Regarding the superior precuneus, this region has reciprocal cortico-cortical connections with the adjacent areas of the posteromedial cortex. This interconnection is bilateral and bridges the homologous components of the two hemispheres (59). Because this region plays a central role in a wide spectrum of higher-order brain functions, increased activation of the precuneus could stimulate the motor cortex, boosting neuroplasticity for motor functional recovery. As a result, motor function in patients with stroke was enhanced and the severity of neurological functional deficits was decreased after 1 month of treatment in the present study. The superior precuneus may be more sensitive to the recovery of brain functions in patients with stroke. Thus, the VMHC in this region was enhanced significantly after treatment and showed no significant difference from that in the healthy controls. The synchrony of spontaneous activity in the bilateral superior precuneus may reflect the treatment effect. Future studies should be designed with multiple time points to determine whether the impaired brain functional connectivity after stroke can be restored to the normal level with treatment intervention.

### Correlations between VMHC and clinical scores after treatment

The primary motor impairments after stroke are spasticity and spastic paresis, which impose substantial challenges to treatment and patient care (60). Manual function dexterity can be described as the ability to perform precise and coordinated hand and finger movements. Accurate evaluation of clinical manual motor function is, therefore, essential for assessing the recovery of motor ability in patients with stroke after treatment. As commonly used clinical recovery assessment scales, the FMA and NDS enable a good overall assessment of arm motor function, the ability to perform simple grasping tasks, and the severity of neurological functional deficits. In the present study, the patients with high changes in FMA or NDS scores between the two time points exhibited motor ability and hand control function that were well-preserved. The severity of stroke damage was reduced after 1 month of treatment. The neurological functional deficits showed recovery. Correspondingly, the patients with high increases in VMHC values in the superior precuneus exhibited enhanced interhemispheric connectivity. The disrupted interhemispheric coupling of the DMN was restored. The precuneus has been suggested to be associated with cognitive impairment in stroke. Significantly enhanced neural interhemispheric coupling in the superior precuneus may indicate that the cognitive impairment of patients was reduced at the second time point. These findings are in accordance with the results of previous studies showing that activation in the precuneus can reflect an increase in functional coupling between bilateral somatosensory areas in patients with stroke (61). The significantly decreased FC in the PCC/precuneus was related to the Montreal Cognitive Assessment scores 10 days after the stroke (8). Spontaneous activity and FC of the PCC/precuneus were correlated with cognitive decline in patients with stroke (16, 42). Precuneus interhemispheric white matter integrity had a positive correlation with haptic performance in normal control participants (62). Combined with these previous studies, the significant correlation results observed in the present study confirmed that the level of coupling between the bilateral superior precuneus can be regarded as a neuroimaging biomarker to reflect the level of recovery of brain function in stroke patients with intervention.

### Functional homotopy in the superior precuneus for the classification

The machine learning results also confirmed that the functional homotopy of the superior precuneus plays an important role in the discrimination of patients with stroke from healthy controls. This finding is important because it demonstrates the specific VMHC changes that occur in patients with stroke. Significantly decreased VMHC values in the important hub of the DMN are generally regarded as a reflection of injury to the brain functional network. A previous study on unilateral ischemic stroke demonstrated that the interhemispheric balance returned to healthy control levels in stroke patients with the successful recovery of dexterous hand function (63). The patient with poor recovery exhibited cerebral blood flow that was lateralized to the contralesional hemisphere, including the regions of SMA, paralimbic anterior cingulate cortex, and superior precuneus. The role of the precuneus cortex in sensorimotor transformations may have some association with goal-directed movements. Another previous study found that precuneus interhemispheric tract integrity can be regarded as a strong predictor of haptic performance (62). Precuneus interhemispheric tracts can be regarded as an appropriate target for piloting rehabilitation to improve poststroke haptic performance. During sensory discrimination, patients with stroke exhibited significantly different cortical activation than controls only in the precuneus (61). The activation in the precuneus may reflect an increase in functional coupling of bilateral somatosensory areas. In the present study, using functional homotopy values in the superior precuneus as a feature achieved high accuracy in the discrimination of stroke participants from healthy controls. Additionally, the correlation results revealed there was a significant relationship between the changes in behavioral performance and the VMHC values in the superior precuneus. Consistent with previous reports and our correlation results, this machine learning result again confirmed the important role of the precuneus in the regulation of brain function in patients with stroke. The VMHC values in superior precuneus can also be considered neuroimaging-based biomarkers for cortical organization in stroke.

The current study has some potential limitations. First, the number of subjects was small, and it is necessary to investigate this topic in a larger sample size. In the present study, we attempted to reduce the effect of a simple size on the results by controlling for the lesion location. This attempt can partially mitigate the influence of sample size. Second, the medication was taken by patients with stroke without placebo control, which may affect the credibility of the present results. Future studies should consider this factor and improve the experimental design.

## Conclusion

In conclusion, the results of the current study demonstrated that restored VMHC of the superior precuneus in patients with stroke can be detected following 1 month of antiplatelet therapy. Importantly, the significant correlations between the VMHC values and the clinical scores in patients with stroke showed that functional homotopy in the superior precuneus can reflect the recovery level of stroke patients' brain function with intervention. The present results have the potential to elucidate the recovery mechanisms of stroke.

## Data availability statement

The original contributions presented in the study are included in the article/Supplementary material, further inquiries can be directed to the corresponding author/s.

## Ethics statement

The studies involving human participants were reviewed and approved by the Ethics Committee of Chengdu University of Traditional Chinese Medicine. The patients/participants provided their written informed consent to participate in this study. Written informed consent was obtained from the individual(s) for the publication of any potentially identifiable images or data included in this article.

## Author contributions

Conceived and designed the experiments: YL and PW. Performed the experiments: ZY and PW. Analyzed the data: YL and XZ. Responsible for patient management and conceptualized the study: JC and PW. Wrote and revised the paper: YL. All authors contributed to the article and approved the submitted version.

## Funding

This research received funding from the National Natural Science Foundation of China (81072864 and 81601483) as well as support from the Medical Science and Technology Research Foundation of Guangdong Province (A2021076). This study was also supported by the Guang Zhou Science and Technology Project (202201011812), the Administration of Traditional Chinese Medicine of Guangdong Province (20221099), the Key-Area Research and Development Program of Guangdong Province (No. 2020B1111100001), and Huang Zhendong Research Fund for Traditional Chinese Medicine of Jinan University.

## Conflict of interest

The authors declare that the research was conducted in the absence of any commercial or financial relationships that could be construed as a potential conflict of interest.

## Publisher's note

All claims expressed in this article are solely those of the authors and do not necessarily represent those of their affiliated organizations, or those of the publisher, the editors and the reviewers. Any product that may be evaluated in this article, or claim that may be made by its manufacturer, is not guaranteed or endorsed by the publisher.

## Supplementary material

Supplementary Figure 1 The symmetrical mask of the default mode network (DMN) and motor network for the voxel-mirrored homotopic connectivity (VMHC) analysis.

Supplementary Figure 2 Classification plot **(A)** and receiver operating characteristic (ROC) curve **(B)** for the comparison between 19 patients with stroke (posttreatment) and 13 healthy controls. The analysis of SVM classification based on the feature of VMHC in the superior precuneus achieved an accuracy of 31.25% (42.86% sensitivity, 9.09% specificity), statistically significant at p < 0.953.
