# Supplementary figures and images for: Aberrant interhemispheric functional reciprocities of the default mode network and motor network in subcortical ischemic stroke patients with motor impairment: A longitudinal study
Source: Front Neurol. 2022 Oct 4;13:996621. doi: 10.3389/fneur.2022.996621 (PMC9577250; doi:10.3389/fneur.2022.996621)

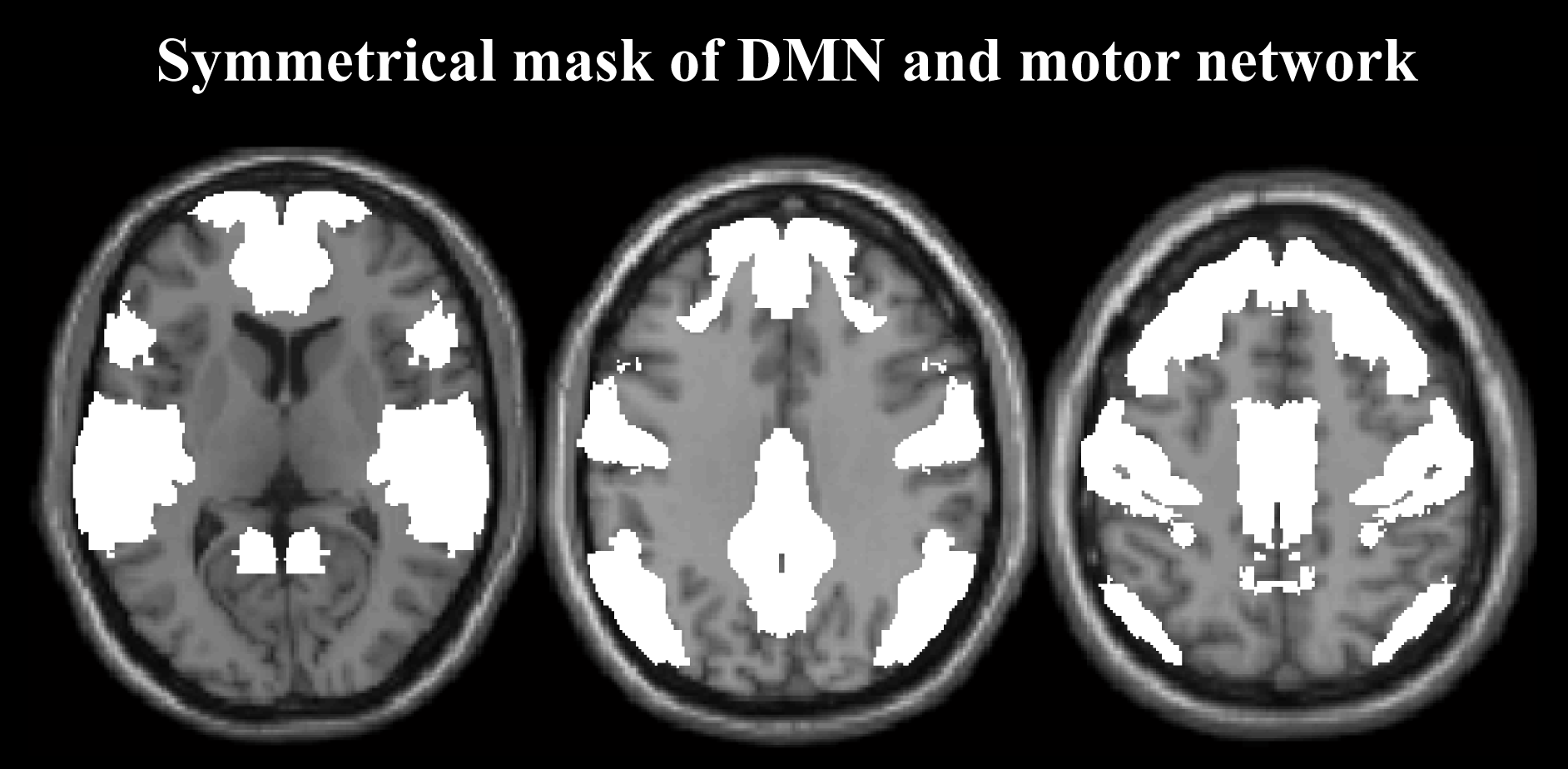

Supplement: Supplementary Figure 1 — The symmetrical mask of the default mode network (DMN) and motor network for the voxel-mirrored homotopic connectivity (VMHC) analysis. [file Image_1.TIF]

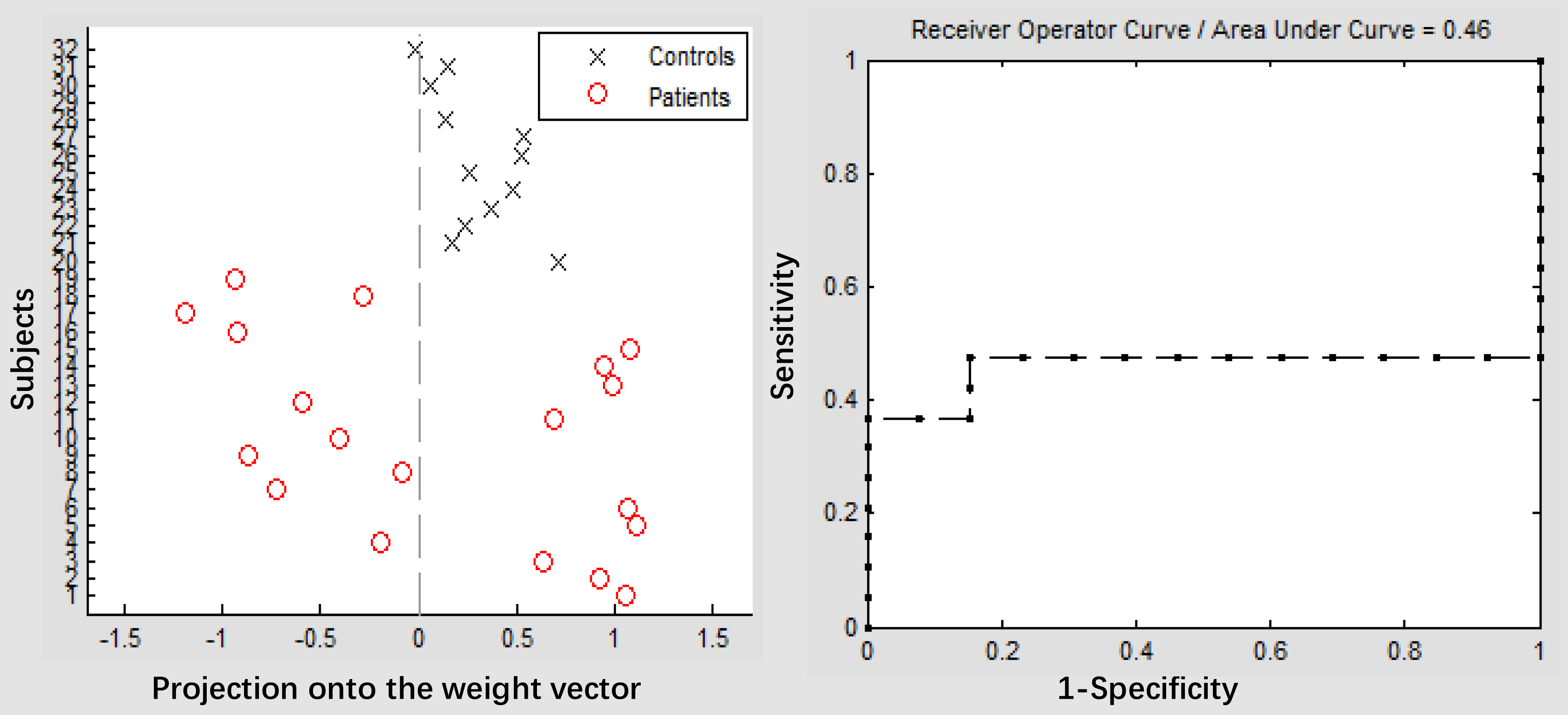

Supplement: Supplementary Figure 2 — Classification plot (A) and receiver operating characteristic (ROC) curve (B) for the comparison between 19 patients with stroke (posttreatment) and 13 healthy controls. The analysis of SVM classification based on the feature of VMHC in the superior precuneus achieved an accuracy of 31.25% (42.86% sensitivity, 9.09% specificity), statistically significant at p < 0.953. [file Image_2.TIF]
